# Analysis of *Plasmodium falciparum* erythrocyte membrane protein 1- DBLα domain variants with respect to antigenic variations and docking interaction analysis with glycosaminoglycans

**DOI:** 10.1186/1475-2875-13-S1-P104

**Published:** 2014-09-22

**Authors:** Deepti Deobagkar, Aarti Ozarkar, Shipra Gupta, Megha Agrawal, Dileep Deobagkar

**Affiliations:** 1 University of Pune, Zoology Department, Pune, India; 2 University of Pune, Bioinformatics Centre, Pune, India

## Background

The variant surface antigen PfEMP1 (*Plasmodium falciparum* erythrocyte membrane protein 1) encoded by the polymorphic multi-copy var gene family plays an important role in parasite biology and the host-parasite interactions. Sequestration and antigenic variation is an essential component in the survival and pathogenesis of *Plasmodium falciparum* and contributes to chronic infection . The DBLα domain of PfEMP1 is a potential target for immuno-epidemiological studies and has been visualized as a vaccine candidate against severe malaria. Extensive *var* gene diversity (DBLα domain of PfEMP1) is an important aspect of pathogenesis in parasite lines. Specific host receptors like heparin, heparan sulphate, blood group A and complement receptor 1 have been reported to bind DBLα domain. Although heparin has been experimentally shown to disrupt the parasite-host interaction and effectively disrupt rosetting, the binding sites for the DBLα domain and mechanism behind heparin-mediated rosette inhibition have not been elucidated.

## Materials and methods

3D structures and epitopes of DBLα domain in 3D7 and in two parasite isolates have been predicted and compared. Docking studies on DBLα domains with human GAG receptors (heparin and heparan sulphate) to predict the strength of association between the protein–ligand interactions were performed.

## Results

The DBLα domain structures showed extensive diversity and polymorphism in their binding sites. The linear as well as conformational epitope prediction analysis predicted several B cell epitopes at variable positions in different DBLα domain variants [1]. The docking results indicate that heparin binds more effectively with high affinity as compared to heparan sulphate with some common interacting residues [2]. These common residues can play an important role in rosetting and will aid in the designing of inhibitors specific to the interactions between DBLα and heparin or heparan sulphate would be important in malaria treatment.

## Conclusions

Thus such an analysis may lead to the development of novel interference strategies to block red blood cell invasion and provide protection against malaria and provide an insight into the complexities of host parasite interactions.
